# 

**Published:** 1902-11-01

**Authors:** 


					?-iiia nvarii^i,. " ine stanuaru 01 nignest purity."?Lancet. inuv. isx, i^.
. CADBURY'S ?a o w CADBURY'S
? COCOA r1& n OOOOA
ABSOLUTELY PURE. JBh DRUGS OR ALKALIES USE*.
mwicH uasMLLXAA
No. 84&-Vol. XXXIII. [a^Newspape"] SATURDAY,
Nov. 1st, 1902.
[SUDtCrsePepn76em8'] TWOPENCE.

				

